# Betaine Alleviated the Ammonia-Induced Apoptosis and Inflammation in the Skin of Largemouth Bass Fed With High-Carbohydrate Diet via Inhibiting MAPK/NFκB-Myd88 Pathway

**DOI:** 10.1155/anu/5681063

**Published:** 2025-10-07

**Authors:** Mingxu Jiang, Jiahong Zou, Huaichi Wang, Ruixuan Zhang, Linyuan Jiang, Yan Lei, Yuhua Zhao, Xiaojuan Cao, Jian Gao, Qingchao Wang

**Affiliations:** ^1^Key Laboratory of Aquacultural Facility Engineering (Ministry of Agriculture and Rural Affairs), College of Fisheries, Huazhong Agricultural University, Wuhan, Hubei 430070, China; ^2^China (Guangxi)-ASEAN Key Laboratory of Comprehensive Exploitation and Utilization of Aquatic Germplasm Resources, Ministry of Agriculture and Rural Affairs, Guangxi Key Laboratory of Aquatic Genetic Breeding and Healthy Aquaculture, Guangxi Academy of Fishery Sciences, Nanning, Guangxi 530021, China

**Keywords:** ammonia stress, betaine, high carbohydrate diet, MAPK, skin

## Abstract

Fish skin provides vital protection against environmental stressors and pathogens, whose health is significantly affected by dietary components. In intensive aquaculture, accumulated ammonia seriously affected fish skin structure and immune responses. In this study, largemouth bass were fed with control diet (Con), high-carbohydrate (HC) diet and HC diet supplemented with betaine (HC + Bet) for 8 weeks before ammonia exposure. The skin structure, immune responses, programmed cell death (PCD), and status of mitogen-activated protein kinase (MAPK) pathways were evaluated. Results indicated ammonia stress increased epidermal thickness and skin mucus cell numbers, while betaine supplementation recovered the HC-restricted epidermal thickness at 7 days post-stress. Ammonia stress also induced the expression of inflammatory cytokines in skin, while betaine significantly inhibited NF-κB/myd88 pathway to alleviate the over-inflammation in skin of HC-fed largemouth bass. Further study identified the significantly increased TdT-mediated dUTP Nick-End Labeling^+^ (TUNEL^+^) cell numbers in bass skin after ammonia stress, which resulted from apoptosis rather than pyroptosis. Furthermore, p38 MAPK and c-Jun N-terminal kinase (JNK) signaling pathways mediated for the ammonia-induced apoptosis, while dietary betaine supplementation inhibited the over-activation of p38 MAPK and JNK pathways to reduce epidermal apoptosis during ammonia stress. Therefore, dietary betaine alleviated both over-inflammation and apoptosis in the skin of largemouth bass fed with HC diet during ammonia stress.

**Summary**

• Ammonia stress significantly damaged skin structure of largemouth bass, which was due to apoptosis rather than pyroptosis.
• Ammonia stress induced over-inflammation in largemouth bass skin via activating NF-κB/Myd88 pathway.
• Betaine alleviated the skin inflammation and apoptosis in largemouth bass fed with high carbohydrate diet via inhibiting MAPK/NF-κB/Myd88 pathway against ammonia stress.

## 1. Introduction

In the past decades, the application of intensive recirculating aquaculture systems (RASs) significantly increased fishery production [1], while the accumulated ammonia and derivative nitrite significantly affected fish immunity and tissue structure, thus threatening the sustainable development of aquaculture [2]. Multiple tissues lesions, including gills, spleen, and liver, have been reported after ammonia stress. Moreover, ammonia stress also induced the inflammatory responses and apoptosis in hybrid grouper (♀*Epinephelus fuscoguttatus* × ♂*E. lanceolatus*) [3]. In yellow catfish (*Pelteobagrus fulvidraco*), ammonia stress upregulated mRNA expression levels of pro-inflammatory cytokines (*il-1*, *il-6*, and *tnf-α*) and inflammatory mediators (*nfκb*, *p65*, and *cox2*) in the head kidney, while downregulating the anti-apoptotic factor *bcl2*, ultimately leading to inflammation and apoptosis [4]. Accordingly, the inflammatory cytokines (*il-1β* and *tnf-α*) in the kidney and pro-apoptotic genes (*caspase3*, *bax*) in the liver of Nile tilapia (*Oreochromis niloticus*) significantly increased after ammonia stress [5]. However, limited research has been conducted on the influence of ammonia stress on skin structure and function, with only one study documenting the heightened presence of skin mucous secretory cells and modified composition of skin mucus microbiota in juvenile yellow catfish following exposure to ammonia stress [6]. In fact, fish owns the ultra-thin, flexible, and lightweight layer skin, which promotes the functions in protection, robustness, and swimming efficiency. Unlike the keratinized skin in terrestrial animals, fish skin is a mucosal surface with a thin epidermis of live cells covered by a mucus layer, and multiple proteins including lysozyme, mucin, transferrin, complement molecules, and heat shock molecules present in skin mucus have extracellular immune functions [7]. Recently, immunoglobulins mainly IgT have been identified in skin mucus to exert immunoprotective roles in defending against pathogenic infection. Thus, fish skin serves as a typical biological barrier to protect organisms from adverse external factors.

The structure and function of animal skin could be manipulated by nutrition, for example, the skin structure of mice was associated with protein intake and carbohydrate intake in males and females [8]. In fish, multiple nutrient composites can also change fish skin structure and mucus composition. Appropriate dietary vitamin C levels could alleviate skin lesion morbidity of young grass carp (*Ctenopharyngodon idella*) after infection with *A. hydrophila* for 14 days [9]. Our former study also identified the protective role of *G. uralensis* and *A. senticosus* extracts supplementation in the skin of yellow catfish during bacterial infection [10]. Betaine, a trimethyl derivative of glycine (N,N,N-trimethylglycine, GB), serves as a methyl donor and osmolyte within cells. Betaine owns the ability to protect cells, proteins, and enzymes from osmotic stress, and it can regulate intracellular fluid concentration and cell volume to maintain osmotic pressure balance in multiple tissues [11]. In animal husbandry, betaine can be used as a dietary supplement to stimulate lipolysis and inhibit fat production, thereby mitigating the negative effects caused by high-carbohydrate (HC) diets, including impacts on growth performance and tissue structure [12]. Furthermore, it can protect gut microbiota from the impact of osmotic changes, enhance nutrient digestion rate, and contribute to maintaining the richness of gut microbial diversity. In chickens, the supplementation of betaine can reduce the rectal temperature and respiratory rate of chickens under heat stress, facilitating the conversion of more expended energy and nutrients into meat [13]. In aquatic animals, betaine is mainly supplied as a feeding stimulant and has been reported to alleviate excessive liver lipid accumulation induced by HC diets in blunt snout bream [14]. Therefore, it is of great importance to explore the regulatory of betaine in the fish skin mucosal system, which would promote the sustainable aquaculture development.

Largemouth bass is an important freshwater aquaculture fish species with high economic value. Dietary inclusion of carbohydrate (mainly starch) is important to produce extruded feed, while over-starch intake beyond 20% would cause multiple adverse effects including restricted growth [15], damaged intestinal structures [16], inflammation, and apoptosis in the liver [17]. Although some studies designed over 30% carbohydrate in largemouth bass feed [15], and others have demonstrated that dietary inclusion of 15% carbohydrate can induce metabolic disorders and tissue inflammation in largemouth bass [18], most studies have identified the negative effects of dietary carbohydrate over 20% [16, 17]. Thus, in our previous study, we designed dietary carbohydrate over 20% as high level, with 12% carbohydrate as control, and also detected the glycogenic liver disease by 20% carbohydrate [19]. Considering the important function of fish skin in defense against ammonia stress and the regulatory role of betaine, the present study added betaine into the HC diet to feed largemouth bass for 8 weeks and then challenged with ammonia stress, with the skin morphology, inflammation, apoptosis, and regulatory pathways detected. The findings of this research aim to provide insights for the mechanism of dietary regulating structure, inflammation, and apoptosis of animal skin and also to optimize aquaculture conditions for the sustainable development of aquaculture practice.

## 2. Materials and Methods

### 2.1. Ethics Statement

The fish rearing experiment and associated procedures have been approved by the Animal Experiment Committee of Huazhong Agricultural University (Approval No. HZAUFI-2017-001).

### 2.2. Feed Formulation, Fish Husbandry, and Sampling

Three isonitrogenous (47.8% crude protein) and isolipidic (11.2% crude lipid) experimental diets were formulated including a control diet (Con), a HC diet, and a HC diet supplemented with betaine (HC + Bet), and feed formulation can refer to our former article [19]. A total of 135 healthy largemouth bass, with an initial body weight of 94.78 ± 1.09 g, were obtained from a standardized aquaculture facility in Ezhou City, Hubei Province, China. After disinfection, the fish were randomly allocated into nine 400-L RASs equipped with temperature control units and a water flow rate of 0.5 L/min. Water quality parameters were maintained as follows: temperature 26 ± 2°C, dissolved oxygen 7.5 ± 0.3 mg/L, pH 7.6 ± 0.2, and natural photoperiod. Fish were fed twice daily at 09:00 and 16:00 for 8 weeks, with feed intake recorded routinely.

The experiment comprised three dietary treatment groups (Con, HC, HC + Bet), each with three replicate tanks (*n* = 3; 15 fish per tank). At the end of the trial, three fish per tank were randomly selected and anesthetized with MS222 (150 mg/L). Concurrently, sterile skin tissue samples (0.5 × 0.5 cm) were excised from the lateral line region below the dorsal fin. A subset of skin samples was fixed in 4% paraformaldehyde, while the remaining tissues were flash-frozen in liquid nitrogen and stored at −80°C for subsequent analyses. Fish growth data have been reported in former article [19]. The remaining fish were allocated for ammonia stress challenge. All procedures adhered to standardized sampling protocols to ensure intergroup comparability.

### 2.3. Ammonia Stress and Sampling

All remaining fish after the sampling at the end of the feeding trial were exposed to ammonia stress under fasting conditions. The ammonia-challenged water was prepared using analytical-grade ammonium chloride (NH_4_Cl, Merck, Whitehouse Station, NJ, USA) as the total ammonia nitrogen (TAN) source. In accordance with established protocols from prior studies [20] and our validated ammonia stress model for largemouth bass (*Micropterus salmoides*) [21], the TAN concentration was rigorously maintained at 13.0 ± 0.5 mg/L through continuous monitoring. Biological sampling was performed at 3 and 7 days post-exposure, with all procedures strictly adhering to the standardized protocols detailed in Section 2.2 to ensure methodological consistency.

### 2.4. Histological Analysis

Skin tissue samples from largemouth bass were subjected to 24-h fixation in 4% neutral buffered formalin. Subsequent processing included dehydration through a graded ethanol series (70%–100%), xylene clearance, and paraffin embedding using standard histological protocols. Serial sections of 5 μm thickness were prepared with a rotary microtome (MICROM International GmbH, Germany) and mounted on glass slides for histochemical analysis.

For cellular characterization, tissue sections underwent dual staining procedures: Alcian Blue-Periodic Acid Schiff (AB-PAS) and Hematoxylin and Eosin (H&E) staining kits (Solarbio, Beijing, China), following manufacturer's specifications. AB-PAS staining specifically differentiated acid mucopolysaccharides in mucus cells through characteristic blue coloration. Histomorphological examination was conducted using an Olympus BX53 light microscope (Japan) equipped with a digital imaging system.

Quantitative analyses were performed using CellSens Standard software (Olympus) with calibrated measurement tools. Epidermal thickness measurements were obtained from digital micrographs using a standardized scale reference system. Mucus cell quantification involved systematic counting of AB-positive cells across five randomly selected microscopic fields (400× magnification) per tissue section, ensuring representative sampling of the epithelial region.

All histological preparations and analytical procedures were conducted under standardized laboratory conditions to ensure methodological consistency. Image acquisition parameters, including magnification, illumination intensity, and exposure time, were maintained constant throughout the study to minimize measurement variability.

### 2.5. TdT-Mediated dUTP Nick-End Labeling (TUNEL) Assay

Apoptotic cells were detected using a one-step TUNEL assay kit (Beyotime Biotechnology, China) according to the manufacturer's protocol. Tissue sections underwent sequential dewaxing in xylene (2 × 5–10 min) followed by dehydration through an ethanol gradient (absolute ethanol for 5 min, 90% ethanol for 2 min, 70% ethanol for 2 min) and distilled water rinsing. After DNase-free Proteinase K treatment (20 μg/mL, Beyotime) at room temperature for 15–30 min, sections were thoroughly washed with PBS (3 × 3 min). The labeling reaction mixture containing terminal deoxynucleotidyl transferase and fluorescein-dUTP was applied to sections in a humidified chamber at 37°C for 60 min, followed by PBS washes to remove unbound reagents. Nuclei were counterstained with anti-fade mounting medium containing DAPI. Fluorescent signals were captured using an Olympus CCD camera-equipped microscope (Japan), with TUNEL-positive cells quantified by systematic random sampling across five nonoverlapping fields per section.

### 2.6. RNA Extraction and RT-qPCR Analysis

Total RNA was extracted from tissue samples using TRIZOL reagent (Magen, Guangzhou, China) with steel bead homogenization following the manufacturer's instructions, followed by RNA purity and concentration assessment via NanoDrop 2000 spectrophotometry (Thermo Fisher, USA) and integrity verification through 1.0% agarose gel electrophoresis. Total RNA (1000 ng) was reverse-transcribed into cDNA using a commercial reverse transcription kit (YEASEN, Shanghai, China), with synthesized cDNA diluted to 200 ng/μL for subsequent RT-qPCR analysis performed on an iCycler iQ5 system (Bio-Rad, USA) employing EvaGreen 2× qPCR MasterMix (ABM, Canada). Thermal cycling conditions included initial denaturation at 95°C for 5 min, 40 cycles of 95°C for 10 s and 60°C for 30 s, with melt curve analysis to confirm amplification specificity. The expression of target genes (primers shown in Table 1) including cytokines-related genes (*il1*β, *il8*, *il10*, *il18*, *tnf-α*, *cxcl10*, *nfkb*, *myd88*, *p65*), immunoglobulins (*igm*, *igt*, *igd*), apoptosis regulators (*caspase3*, *caspase8*, *caspase9*, *bax*, *bcl2*, *p53*), and pyroptosis-associated genes (*gsdme*, *asc*, *nlrp3*) was analyzed via normalized to the endogenous control *18s* rRNA using the 2^−ΔΔCt^ method. Statistical significance was determined through GraphPad Prism 6 and SPSS software with appropriate post hoc analyses.

### 2.7. Western Blotting Analysis

Skin tissue samples from different groups were homogenized in RIPA lysis buffer supplemented with protease and phosphatase inhibitors to extract total proteins. Protein concentrations were determined using an Enhanced BCA Protein Assay Kit (P0010, Beyotime, China) and adjusted to equal concentrations across groups. Equal amounts of protein were separated by SDS-PAGE and transferred to PVDF membranes. After blocking with 5% skim milk for 2 h at room temperature, the membranes were incubated overnight at 4°C with primary antibodies.

Subsequently, membranes were incubated with HRP-conjugated secondary antibodies for 1 h at room temperature. Immunoreactive bands were visualized using ECL chemiluminescent reagent (GE Healthcare, USA) on an Amersham Imager 600 system, and protein expression levels were quantified with ImageQuant TL software. Primary antibodies against p-c-Jun N-terminal Kinase (JNK), p38 mitogen-activated protein kinase (MAPK), and p-ERK1/2 were obtained from Cell Signaling Technology (USA), while β-actin antibody was purchased from ABClonal Biotechnology (Wuhan, China).

### 2.8. Statistical Analysis

The experimental data were analyzed using the SPSS statistical software. The results are presented in the form of mean ± standard deviation. Apart from protein level results, all data were checked for normality using the Shapiro–Wilk's W test. Data that exhibited normal distribution underwent two-way analysis of variance (factorial ANOVA) to determine the main effects of diet and ammonia stress on gene expression and their interactions. In cases where a significant interaction between diet and ammonia stress was observed, data were subjected to one-way ANOVA followed by Tukey's post hoc multiple comparison test to examine differences among all treatments.

Protein level data underwent one-way ANOVA and Tukey's post hoc multiple comparison test to analyze the significance of differences. The significance level was set at *p* < 0.05, indicating the presence of statistically significant differences.

## 3. Results

### 3.1. Betaine Alleviated the Ammonia Stress-Induced Skin Damage in Largemouth Bass Fed With HC Diet

**Figure 1:**
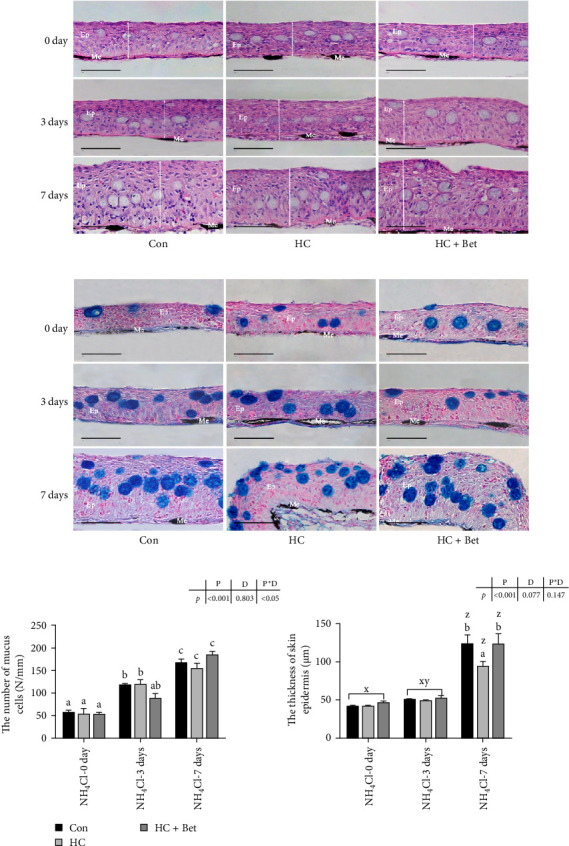
Effects of betaine supplementation on the skin structure of largemouth bass fed with high carbohydrate diet against ammonia stress. (a) H&E staining of largemouth bass skin fed with three diets at 0, 3, and 7 days after ammonia stress. Scale: 50 μm. (b) A&B staining of largemouth bass skin fed with three diets at 0, 3, and 7 days after ammonia stress. Scale: 50 μm. (c) Statistical analysis of the thickness of skin epidermis and the number of mucus cells in largemouth bass fed with three diets at 0, 3, and 7 days after ammonia stress. All data were analyzed with two-way ANOVA to determine the main effects of ammonia stress period (P) and diet (D) and their interactions. Data are expressed as means ± SEM (*n* = 6). Mean values with different letters indicated significant difference among groups, *p* < 0.05. Ep, epidermis; Me, melanophores.

As shown in Figure 1, with the multilayer structure composed of epidermis, dermis, and basement membrane, largemouth bass skin functions as a key biological barrier in defending against multiple stresses. No noticeable differences were detected in skin structure among the three dietary groups at the end of the 8-week feeding trial. However, ammonia stress significantly altered the largemouth bass skin structure, as the epidermal layer was much thicker in 3-day ammonia stress group and further aggravated after ammonia stress for 7 days (Figure 1a,c). Furthermore, the quantity of mucous cells in bass skin also significantly increased after ammonia stress, which further accelerated with the extended stress period (Figure 1b,c). Fish skin structure in the HC group exhibited similar changes to the Con group after ammonia stress for 3 days, and no notable variance was detected in the quantity of mucous cells between two groups. However, after ammonia stress for 7 days, the skin thickness in the HC group was significantly lower than that of the Con group. On the other hand, after ammonia stress for 7 days, dietary betaine supplementation recovered the skin histological structure of fish fed with HC diet, as the skin epidermal thickness in the HC + Bet group showed no significant difference from that in the Con group. No significant differences were observed in the number of mucous cells in fish skin among three groups.

### 3.2. Betaine Alleviated the Ammonia Stress-Induced Inflammation in Skin of Largemouth Bass Skin Fed With HC Diet

**Figure 2:**
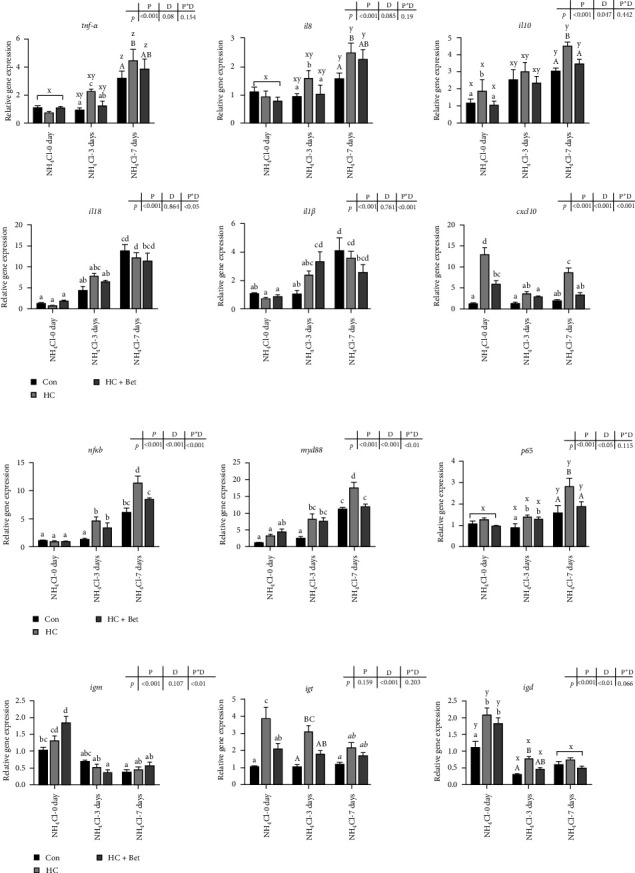
Effects of betaine supplementation on the expression of genes involved in (a) inflammation (tnfα, *il8*, *il10*, *il18*, *il1β*, *cxcl10*), (b) NFκB/Myd88 pathway (*nfκb*, *myd88*, *p65*), and (c) immunoglobulins (*igm*, *igd*, *igt*) in the skin of largemouth bass fed with high-carbohydrate diet against ammonia stress. All data were analyzed with two-way ANOVA to determine the main effects of ammonia stress period (P) and diet (D) and their interactions. Data are expressed means ± SEM (*n* = 6). Mean values with different letters indicated significant difference among groups, *p* < 0.05.

As shown in Figure 2, the expression levels of tumor necrosis factor-α (*tnf-α*), interleukin1*β* (*il1β*), *il8*, *il10*, and *il18* in the skin of largemouth bass were significantly increased after ammonia stress and further increased with prolonged ammonia stress period. Accordingly, the expression of nuclear factor-κB (*nf-κb*), myeloid differentiation factor (*myd88*), and *p65* in all three groups also increased significantly after ammonia nitrogen stress. On the other hand, the expression levels of cxc chemokine 10 (*cxcl10*) showed no significant change and even decreased after ammonia stress. Furthermore, following 7 days of exposure to ammonia stress, the levels of *nf-κb* and *myd88* expression in the largemouth bass skin of the HC group were markedly elevated compared to those in the Con group, while betaine supplementation significantly decreased the expressions of these two genes in skin induced by HC.

Besides the cytokines, immunoglobulins have also been reported to exist in fish skin mucosa; thus, the expression of three immunoglobulins was also evaluated via RT-qPCR. As shown in Figure 2c, the expressions of *igt* and *igd* in the largemouth bass skin of the HC group were significantly higher than those of the Con group, while the expression of *igm* in the HC + Bet group was significantly higher than those of Con group. Ammonia stress significantly inhibited the expression levels of *igm* and *igd* in largemouth bass skin in three groups. On the other hand, the expression of *igt* in the Con group showed no significant changes after ammonia stress. However, betaine supplementation significantly decreased the expression of *igt* induced by HC diet and also showed no significant changes after ammonia stress.

### 3.3. Betaine Alleviated the Ammonia Stress-Induced Apoptosis in Skin of Largemouth Bass Skin Fed With HC Diet

**Figure 3:**
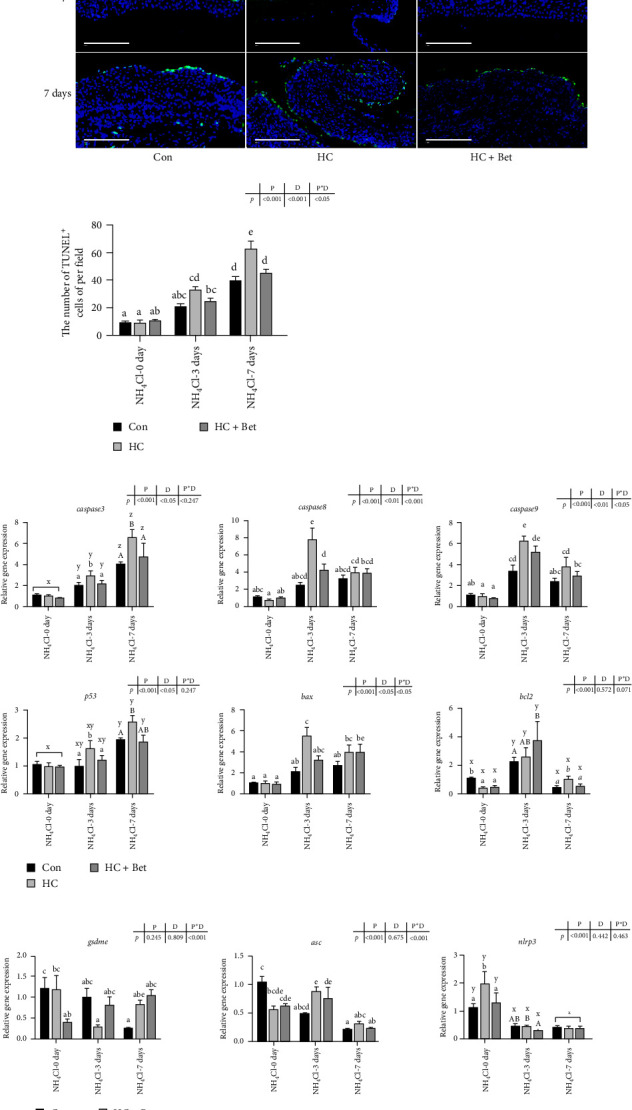
Effects of betaine supplementation on programmed cell death of largemouth bass skin fed with high-carbohydrate diet against ammonia stress. (a) TUNEL assay of largemouth bass skin fed with three diets at 0, 3, and 7 days after ammonia stress. Scale: 100 μm. (b, c) The relative mRNA expression of genes involved in apoptosis (*caspase3*, *caspase8*, *caspase9*, *p53*, *bax*, *bcl2*) and pyroptosis (*gsdme*, *asc*, *nlrp3*) in largemouth bass skin fed with three diets at 0, 3, and 7 days after ammonia stress. All data were analyzed with two-way ANOVA to determine the main effects of ammonia stress period (P) and diet (D) and their interactions. Data are expressed as means ± SEM (*n* = 6). Mean values with different letters indicated significant difference among groups, *p* < 0.05.

TUNEL assay was used to further analyze the programmed cell death (PCD) in the skin of largemouth bass after ammonia stress. As shown in Figure 3a, the TUNEL^+^ cells in largemouth bass skin are predominantly distributed in the epidermal layer, and no significant variation was detected in the number of TUNEL^+^ cells among three dietary groups. Ammonia stress significantly increased the number of TUNEL^+^ cells in the epidermal layer, which further accelerated with the extended ammonia stress period. After ammonia stress for 3 days, more TUNEL^+^ cells were detected in the skin of largemouth bass in the HC group than those in the Con group. However, betaine supplementation significantly alleviated the increased number of TUNEL^+^ cells induced by HC diet. Moreover, after ammonia stress for 7 days, the number of TUNEL^+^ cells in the HC + Bet group showed no significant difference with that in the Con group (Figure 3a).

Considering TUNEL^+^ cells may result mainly from both apoptosis and pyroptosis, the mRNA expression levels of genes related to these two types of PCD in largemouth bass skin were analyzed using RT-qPCR. As shown in Figure 3b, the expression of *caspase3*, *caspase8*, and *caspase9*, which are executors for apoptosis, increased after ammonia stress, and *caspase3* showed the highest expression level after ammonia stress for 7 days. Accordingly, the expression of *p53* and *bax*, which plays the pro-apoptotic role, also increased after ammonia stress. Moreover, the expression of these genes in the HC group was higher than those in the Con group after ammonia stress. However, betaine supplementation significantly decreased the HC-induced higher expression of these apoptotic genes after ammonia stress. The expression of *bcl2* was significantly increased after 3 days of ammonia stress but then decreased after 7 days of ammonia stress. After 3 days of ammonia stress, the expression level of *asc* in the skin of the HC group of largemouth bass was higher compared to the control group. However, there were no significant differences in the expression of *gsdme* and *nlrp3* among the three groups before and after ammonia stress. These results indicated that the ammonia stress-induced PCD in largemouth bass skin mainly results from apoptosis.

### 3.4. p38 MAPK and JNK Signaling Pathways Integrated the Betaine-Regulatory Role in Inflammation and Apoptosis of Largemouth Bass Skin After Ammonia Stress

**Figure 4:**
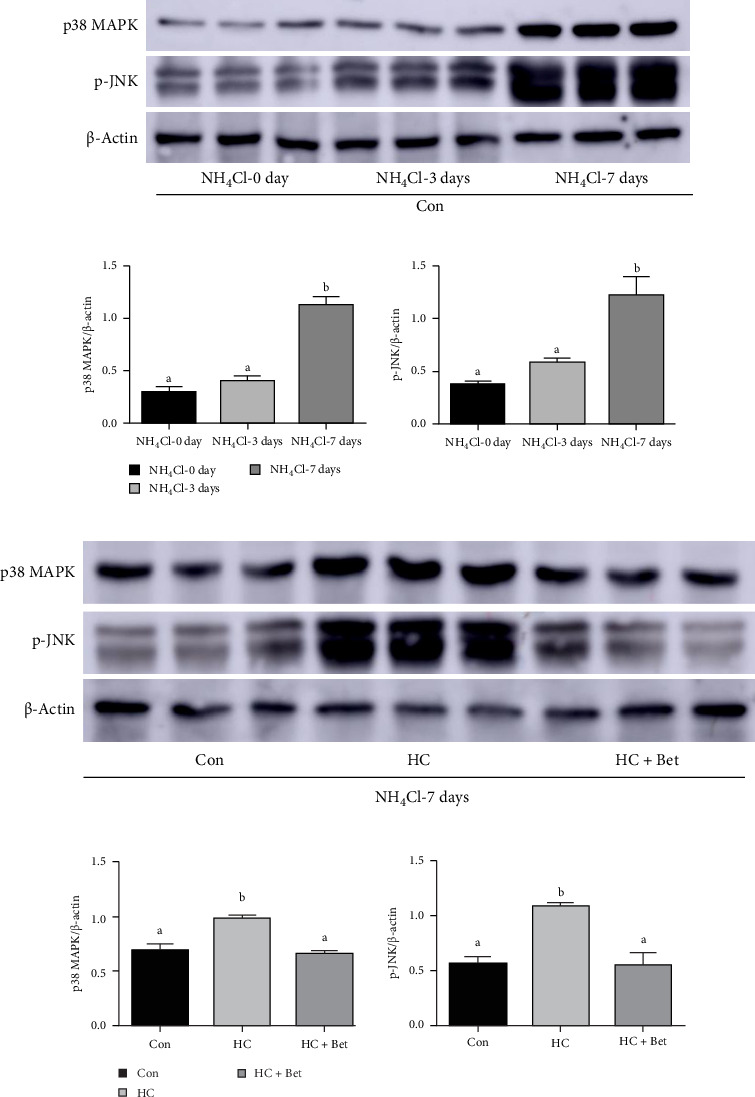
Effects of betaine supplementation on MAPK signaling pathway of largemouth bass skin fed with high-carbohydrate diet against ammonia stress. (a) Western blotting of p38 MAPK and p-JNK protein level in largemouth bass skin at 0, 3, and 7 days after ammonia stress. (b) Statistical analysis of the ratio of p38 MAPK/β-actin and p-JNK/β-actin (*n* = 3). (c) Western blotting of p38 MAPK and p-JNK proteins in largemouth bass skin fed with three diets at 7 days after ammonia stress. (d) Statistical analysis of p38 MAPK/β-actin and p-JNK /β-actin ratio at 7 days after ammonia stress. All data were analyzed with one-way ANOVA and data were mean ± SEM (*n* = 3). Mean values with different letters indicated significant difference among groups, *p* < 0.05.

To explore the regulatory mechanism of apoptosis in largemouth bass skin after ammonia stress, the activation status of three MAPK pathways including p38 MAPK, phosphorylated JNK (p-JNK), and ERK1/2 was assessed using western blotting analysis. As shown in Figure 4a,b, the protein levels of both p38 MAPK and p-JNK in largemouth bass skin of the Con group showed a trend to increase with the prolonged ammonia stress period, and their expression levels were significantly higher in the groups of ammonia stress for 7 days than those in the groups before ammonia stress. However, the protein levels of ERK1/2 showed no significant changes after ammonia stress (Supporting Information Figure S1). In order to further investigate the influence of dietary carbohydrate level and betaine supplementation on the MAPK signaling pathways after ammonia stress, a systematic comparison was conducted on the levels of p38 MAPK and p-JNK in the skin of largemouth bass in the Con, HC, and HC + Bet groups at 7 day after ammonia stress. According to the data presented in Figure 4c,d, the HC group demonstrated notably increased protein levels of p38 MAPK and p-JNK compared to the Con group. However, the addition of dietary betaine effectively inhibited the elevated protein levels of p38 MAPK and p-JNK triggered by a HC diet and their expression levels in the HC + Bet group were comparable to those observed in the Con group after ammonia stress for 7 days.

## 4. Discussion

Among all water parameters, the enriched ammonia in the intensive aquaculture system can permeate fish mucosal tissues including gill, skin, and other tissues, thus affecting fish health and inducing multiple fish diseases. Previous studies have shown that ammonia stress induced gill hyperplasia and a marked expansion in gill filament spacing of largemouth bass [20]. Besides gill, the fish skin, a tri-layered structure comprising epidermis, dermis, and hypodermis, plays vital physiological roles in nutrient absorption, cutaneous gas exchange, ion transport, and nitrogenous waste excretion, alongside its protective function. In this study, clear epidermal layer and melanocytes were detected in the skin of largemouth bass fed with three diets, which is similar to the skin morphology of bony fish in previous studies [22]. However, ammonia stress significantly changed largemouth bass skin structure, as the thickness of skin in bass significantly increased after ammonia stress for 7 days (Figure 1b,c). The increased skin thickness might contribute to the adaptive defense against water-borne stress including ammonia in the present study. In fact, the skin epidermis of *Seriola dumerili* thinned markedly after *Neobenedenia girellae* infection when reared at 25 and 30 °C [23]. Moreover, in African dipnoi (*Protopterus* sp.), short-term aestivation induces major reorganization, with narrowing of epidermal layers and decrease of mucous cells [24]. Dietary manipulation has been reported to sustain fish skin structure against environmental stress and pathogenic infection. For example, in goldfish (*Carassius auratus*), dietary cMOS supplementation increased the thickness of dermal dense layer of fish skin both before and after 14 days of *Ichthyophthirius multifiliis* challenge [25]. In our study, although the thickness of bass skin in the HC group also significantly increased after ammonia stress, its thickness after ammonia stress for 7 dpi was significantly less than that in the Con group. Thus, the HC diet resulted in the weaker skin morphology to defend against ammonia challenge. Dietary betaine supplementation significantly increased the thickness of skin in the HC + Bet group with a similar level to that in Con group, indicating that betaine addition significantly mitigates the adverse effects of HC diet on fish skin during ammonia challenge. Besides skin dermal layer thickness, the number and distribution of fish skin mucus cells are affected by many adverse biological and abiotic environmental factors. The fish epidermis contains three distinct types of mucus-secreting cells in addition to epithelial cells: goblet cells, sacciform cells, and club cells [26]. Previous research has indicated that monitoring the number of skin mucus cells in fish can serve as a reliable method to assess stress levels impacting fish survival [27]. The introduction of chicken manure into the aquaculture water initially resulted in a sharp reduction in the quantity of mucous cells in the skin of *Cyprinus carpio* [28]. In our study, the number of mucous cells in fish skin of three groups increased significantly after 7 days of ammonia stress, and no significant difference was found among three groups. This is the same as the results of studies with *Dicentrarchus labrax*, which also exhibited a significant increase in the number of skin mucous cells during low oxygen and high concentrations of nitrate challenge [27].

As mentioned above, fish skin mucous layer also contains many important proteins and enzymes, such as proteases, antimicrobial peptides (AMPs), lectins, lysozymes, immunoglobulins, complement proteins, and transferrin, which play an important role in the fish immune system [29]. Previous studies have demonstrated that ammonia can impact fish immune response by modulating cytokines and engaging in the pro-inflammatory responses in multiple fish organs including gill and liver. For example, high ammonia concentrations increased the expression of *tnf-α* and *il1β* in the gills of *Trachinotus ovatus* via activating *hif-1α*/*nfκb* signaling pathway [30]. Moreover, the expression of *il1β* and *cox2* in the gills of *Pelteobagrus fulvidraco* also increased after ammonia stress, along with the upregulated NF-κB and MAPK pathway [31]. In this study, the mRNA expressions of inflammatory cytokines including *il1β*, *il18*, and *tnf-α* significantly increased after ammonia stress, which are accompanied by the increased expression of *nfκb* and *myd88* after 7 days of ammonia stress. Therefore, the nfκb/myd88 signaling pathway was triggered by ammonia stress to promote the secretion of pro-inflammatory cytokines in mucosal tissues. Similar to tissue structure, the inflammatory responses could also be affected by dietary manipulation. Multiple studies have indicated that HC diets exhibited the pro-inflammatory effects. For instance, HC diet leads to inflammation and cell apoptosis in the intestinal epithelial cells of largemouth bass [32]. In our study, HC diet feeding for 8 weeks resulted in a significant increase in the expression of the pro-inflammatory chemokine *cxcl10*, whose expression was also significantly higher than that in the other two groups after ammonia stress. After 7 days of exposure to ammonia stress, the HC group exhibited significantly elevated levels of pro-inflammatory cytokines, such as *tnf-α*, *il8*, and *il10*, in comparison to the Con group. However, dietary betaine supplementation significantly inhibited the expression of *nfκb*/*myd88* to alleviate the ammonia-induced inflammation of skin in fish fed with HC diet. All these results indicated that betaine plays a protective role in suppressing skin inflammation. Early studies showed that dietary betaine decreased the inflammatory response in *Vibrio anguillarum*-infected ayu (*Plecoglossus altivelis*) tissues and monocytes/macrophages [33]. Besides the innate immune defense system, fish also develops the adaptive immune defense systems to protect against various challenges. Especially, the maintenance of adequate immunoglobulins in mucosal tissues and systematic immune organs is key to fight against pathogenic infection. In our present study, ammonia stress significantly decreased the mRNA expression levels of *igm* and *igd* in bass skin, indicating that ammonia stress suppressed the adaptive immune system, which may increase the risk of pathogenic infections. This is similar to previous studies in red seabream (*Pagrus major*) [34], as ammonia stress significantly decreased their levels of total Ig (immunoglobulin). Moreover, ammonia exposure also resulted in a significant decrease in *igm* levels in the spleen of blunt snout bream (*Megalobrama amblycephala*) [35]. Recent studies have also identified the key roles of IgT in fish skin against parasite [36] and bacterial [37] infections, while ammonia stress showed no significant effect on the mRNA expression of *igt* in bass skin in our study. On the other hand, HC induced the expression of *igt* in skin, while betaine significantly alleviated such upregulation. Further studies with specific antibodies against bass IgT should be conducted to evaluate the protein levels and titer of Igs, including IgM and IgT, in the skin of bass fed with different diets after ammonia stress.

Besides the immune responses, PCD, which acts as an essential and complex biological process in eukaryotes, could also be elicited by environmental stress. The terminal deoxynucleotidyl TUNEL assay is a well-established method employed for identifying DNA fragmentation associated with cell death, specifically the presence of 3′-OH DNA termini resulting from endonuclease activity. In our study, ammonia stress for 7 days resulted in a notable rise in the quantity of TUNEL^+^ cells, indicating that ammonia stress can indeed trigger PCD in largemouth bass skin. Multiple PCD types have been identified in recent years including apoptosis, necroptosis, pyroptosis, ferroptosis, and autophagy [38]. Thus, in order to further investigate the specific PCD type during ammonia stress, RT-qPCR was performed to assess the mRNA levels of genes involved in pyroptosis and apoptosis, which both could be detected by TUNEL. Our study indicated that the expression levels of pyroptosis-related genes, including NOD-like receptor pyrin domain-containing protein 3 (*nlrp3*), apoptosis-associated speck-like protein containing a CARD (*asc*), and Gasdermin family protein-E (*gsdme*), were not significantly increased in the largemouth bass skin after ammonia stress and even decreased in certain groups. On the other hand, the expression levels of *caspase3*, *caspase8*, *caspase9*, and *bax*, which are involved in apoptosis, were significantly increased at various time points following ammonia nitrogen stress. This is similar to the reports in gill, as ammonia stress induced the significant expression of *caspase3*, thereby activating the cellular apoptosis system in *Megalobrama amblycephala* [39]. Previous study in *Lateolabrax japonicus* showed that ammonia nitrogen stress induced hepatic apoptosis via upregulating the expression levels of *p53*, *caspase3*, *caspase9*, and *bax* [40]. In accordance with previous results, dietary components can also affect cell apoptosis. For example, low-protein high-starch diet has been reported to induce liver inflammation and apoptosis in juvenile largemouth bass *Micropterus salmoides* [19]. Here, in our study, the number of apoptotic cells of largemouth bass skin in the HC group after ammonia nitrogen stress was significantly higher than that in the Con group, suggesting that HC diet can exacerbate ammonia nitrogen stress-induced apoptosis. However, TUNEL^+^ cells in fish skin of the HC + Bet group after ammonia stress for 7 days showed no significant difference with that in the Con group. In fact, many reports have reported the inhibitory role of dietary betaine on oxidative stress and apoptosis. Dietary betaine significantly attenuated HC-diet-induced oxidative stress, endoplasmic reticulum stress, and apoptosis of mandarin fish (*Siniperca chuatsi*) [41].

In order to further illustrate the regulatory mechanism of apoptosis and inflammation in fish skin under conditions of ammonia stress, the activation status of MAPK pathways, which translate extracellular stimuli into a variety of physiological and pathological processes, was evaluated. The p38 MAPK and JNK signaling pathways exhibit primary activation in response to diverse stressors, intricately connected to apoptosis initiation through transcriptional reprogramming and post-translational modifications. Conversely, ERK1/2 cascades predominantly facilitate mitogen-induced signal transduction, typically playing a cytoprotective role, although context-specific pro-apoptotic effects have been observed in certain cellular environments [42]. In fish, heat stress, high salinity, low oxygen, and bacterial infection can promote MAPK signaling and stimulate the body to produce immune responses in tilapia [43]. It has also been reported that ammonia stress stimulates immune responses by activating MAPK activity. In this study, the protein level of both p38 MAPK and JNK significantly increased after ammonia stress for 7 days. Previous studies have reported that oxidative stress and inflammation can activate the p38 MAPK signaling pathway through phosphorylation of p38 kinase, and the activation of p38 MAPK signaling pathway can continue to induce apoptosis [44], which is consistent with our experimental results with the activated p38 MAPK during ammonia stress. Moreover, ROS accumulation induced by ammonia nitrogen stress can mediate the activation of JNK signaling pathway, and JNK pathway inhibition can prevent some of the apoptosis in zebrafish triggered by BDE-47 treatment, suggesting the contributor role of JNK to apoptosis [45]. These results also supported the activated JNK signaling pathway in fish skin during ammonia stress, indicated by the increased protein level of p-JNK. Furthermore, MAPK pathways and apoptosis in fish were also reported to be regulated by diet, for example, dietary vitamin D supplementation attenuated cell apoptosis in the skin of grass carp (*Ctenopharyngodon idella*) challenged with *Aeromonas hydrophila* by decreasing the mRNA and protein levels of apoptosis factors involved in death receptor and mitochondrial pathway processes related to p38 MAPK and JNK signaling [46]. Here, in our study, HC diet even exacerbated the activation of MAPK signaling pathway proteins in largemouth bass skin under 7-day ammonia stress. Similar results were observed in studies of Nile tilapia, where a HC diet reduced their resistance to pathogens and increased the activation level of p38 MAPK [47]. On the other hand, betaine addition significantly alleviated the effects on overactivated p38 MAPK and JNK pathways induced by HC diet, thereby reducing the excessive apoptotic reaction of fish skin epidermal cells. In juvenile black seabream (*Acanthopagrus schlegelii*), studies have also shown that betaine can regulate the balance of Srebp-1 and Pparɑ by activating the Sirt1 signaling pathway, improving hepatic lipid metabolism abnormalities, and exerting a significant protective effect on the liver [48].

## 5. Conclusion

Thus, our study indicated that ammonia exposure significantly damaged fish skin structure and induced inflammation and apoptosis via activating p38 MAPK and JNK pathways. Dietary betaine supplementation significantly alleviated the skin lesion of largemouth bass fed with HC diet during ammonia exposure and also exhibited the regulatory role in skin inflammatory responses via regulating the MAPK/NFκB-Myd88 pathway.

## Figures and Tables

**Table 1:** Primers used in the present study.

Gene name	Abbreviation	Primer sequence	Tm (°C)
18S ribosomal RNA	*18s*	F:GCAAAGCTGAAACTTAAAGGAATTG	61.5
		R: TCCCGTGTTGAGTCAAATTAAGC	61.2
caspase3	*casp3*	F: GCTTCATTCGTCTGTGTTC	50.3
		R: CGAAAAAGTGATGTGAGGTA	50.9
caspase8	*casp8*	F: GAGACAGACAGCAGACAACCA	55.4
		R: TTCCATTTCAGCAAACACATC	56.0
caspase9	*casp9*	F: CTGGAATGCCTTCAGGAGACGGG	68.9
		R: GGGAGGGGCAAGACAACAGGGTG	70.6
Tumor protein p53	*p53*	F: TGTATCCCAGCGTGTTTTGGA	61.3
		R: GTTTCAGTGCGTTCAGTGCC	58.8
Tumor necrosis factor-α	*tnf-α*	F: GCATACCCAGAATGTGAGA	50.6
		R: CATAACCGCCACGACTAA	52.3
bcl2-associated x	*bax*	F: ACTTTGGATTACCTGCGGGA	59.6
		R: TGCCAGAAATCAGGAGCAGA	58.9
b-cell lymphoma-2	*bcl2*	F: CGCCATCCACAGAGTCCT	55.7
		R: CCGGAACAGTTCGTCTATCACC	61.1
NACHT, LRR, and PYD domains-containing protein 3-like	*nlrp3*	F: TCAATCACGCAAAGTATGG	52.2
		R: TTCCATTTCAGCAAACACATC	56.0
Apoptosis-associated speck-like protein containing a CARD	*asc*	F: AAAGGCAATAGCAGACGC	53.6
		R: AAGTGGAAACCAGGATGT	48.3
Gasdermin-e	*gsdme*	F: TTACTTGGTATCTGGTGGTG	50.5
		R: ACATTCATCTGTCGTGGC	50.7
Interleukin-18	*il18*	F: TTTTTCCCCAGGTCTTCCTGATGG	67.4
		R: GTGTCCTCATCCCAATCTGTTTCA	62.8
Interleukin 1, beta	*il1β*	F: TCAGCCACGGAGGAAAAAGAC	61.7
		R: ACCTACATCAGGTGAGGTCTCTAA	56.9
Interleukin-8	*il8*	F: TGGCCATCAGTGAAGGGATG	61.2
		R: CAGTGGGAGTTGGCAGGAAT	59.6
Interleukin-10	*il10*	F: TTCAAAAGCCCGTTTGCCTG	63.5
		R: AGCTCGTCGAAGATCTGCTG	57.9
Nuclear factor-κ-gene binding	*nf*-κB	F: CCACTCAGGTGTTGGAGCTT	57.3
		R: TCCAGAGCACGACACACTTC	56.2
relA proto-oncogene, NF-κB subunit	*p65*	F: CCCCTCACTGCTCCTCTACT	55.7
		R: GTGCGATGTCAATCATGCCC	61.6
Myeloid differentiation factor 88	*myd88*	F: CTCAACCCCAAGAACACA	51.0
		R: CGAAGATCCTCCACAATG	50.7
Immunoglobulin M	*igm*	F: CTCAATGACCCCCCCTAA	55.1
		R: CAAGCCAAGACACCAAAA	51.9
Immunoglobulin T	*igt*	F: GAAGGTCAACAACGCTGAGTG	58.1
		R: TGTTGCTGGTCACATCTAGTCC	57.5
Immunoglobulin D	*igd*	F: AAGGGAAACAGTGCTGTGCT	57.2
		R: TTGCCAGTGGGGTTTGACTT	60.2

## Data Availability

Data are available to be got from corresponding author under necessary request.

## Nomenclature

ASC: Apoptosis-associated speck-like protein containing a CARD
Bax: Bcl-2–associated X protein
Bcl2: B cell lymphoma 2
Bet: Betaine
Con: Control
Cxcl10: CXC chemokine ligand-10
Cxcr3: CXC chemokine receptor 3
ERK: Extracellular regulated protein kinases
GSDME: Gasdermin family protein-E
HC: High-carbohydrate
Ig: Immunoglobulin
IL: Interleukin
JNK: c-Jun N-terminal kinase
MAPK: Mitogen-activated protein kinase
Myd88: Myeloid differentiation primary response gene 8
NLRP3: NOD-like receptor pyrin domain-containing protein 3
PCD: programmed cell death
TUNEL: TdT-mediated dUTP Nick-End Labeling
TNF: Tumor necrosis factor.

## Supporting Information

Supporting Information Figure S1: Effects of betaine supplementation on ERK1/2 signaling pathway in the skin of largemouth bass fed with high carbohydrate diet against ammonia stress.
