# Supplementary material for: Betaine Alleviated the Ammonia-Induced Apoptosis and Inflammation in the Skin of Largemouth Bass Fed With High-Carbohydrate Diet via Inhibiting MAPK/NFκB-Myd88 Pathway
Source: Aquac Nutr. 2025 Oct 7;2025:5681063. doi: 10.1155/anu/5681063 (PMC12520811; doi:10.1155/anu/5681063)
Supplement: Supporting Information — Figure S1: Effects of betaine supplementation on ERK1/2 signaling pathway in the skin of largemouth bass fed with high carbohydrate diet against ammonia stress. [file 5681063.f1.docx]

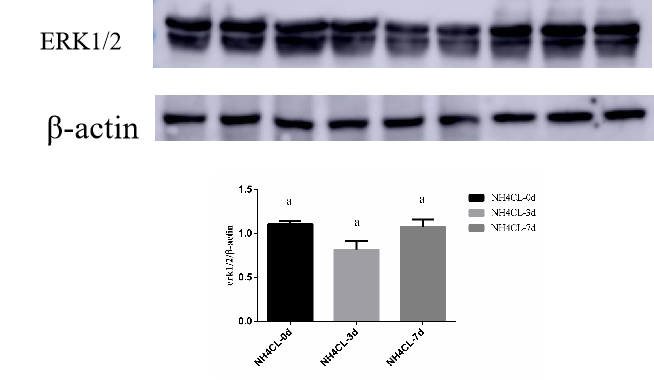


Supplenmental Fig. 1 Effects of betaine supplementation on ERK1/2 signaling pathway in the skin of largemouth bass fed with high carbohydrate diet against ammonia stress. All data were analyzed with one-way ANOVA and data were mean ± SEM (n = 3). Mean values with same letters indicated non-significant difference among groups, *p* < 0.05.
